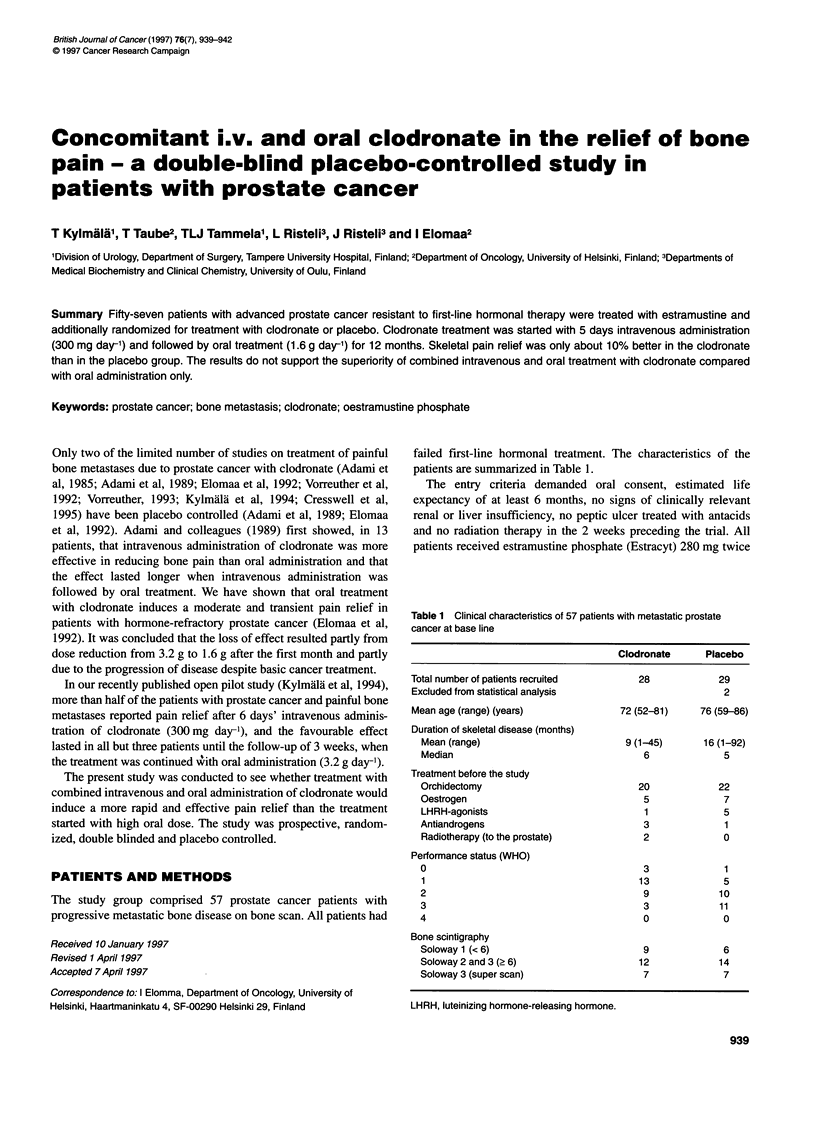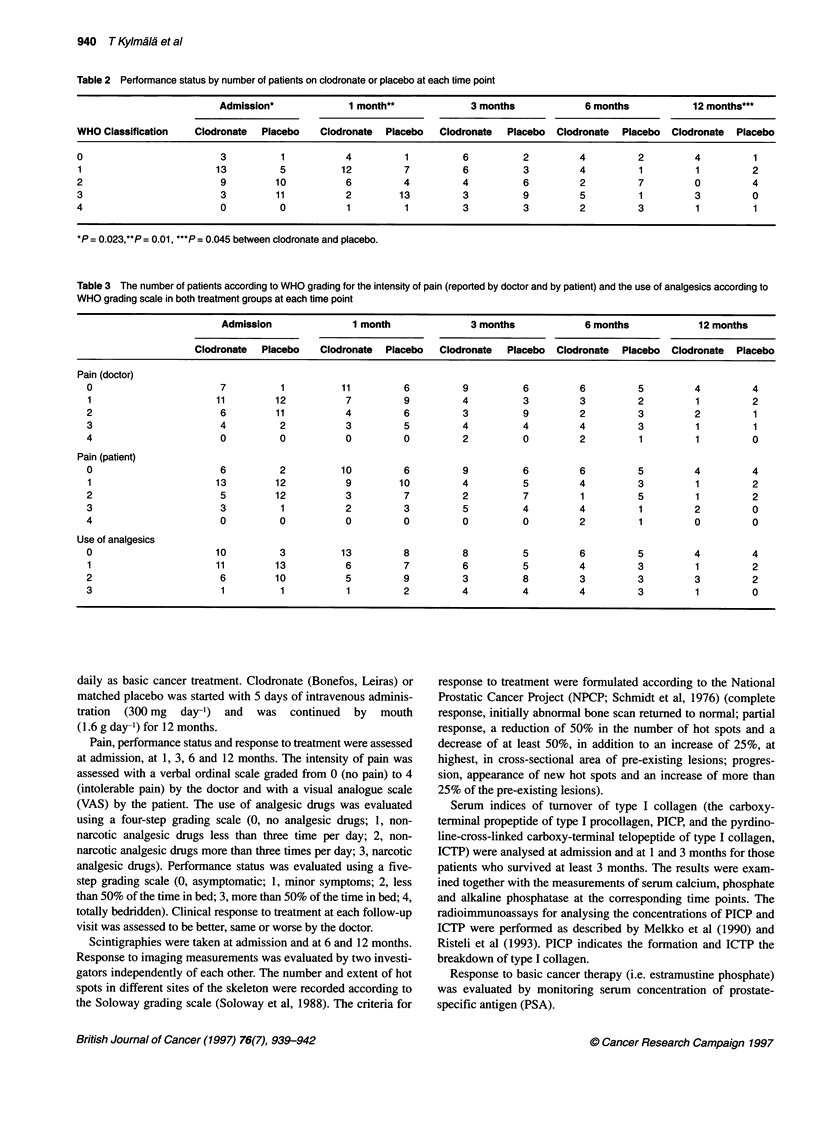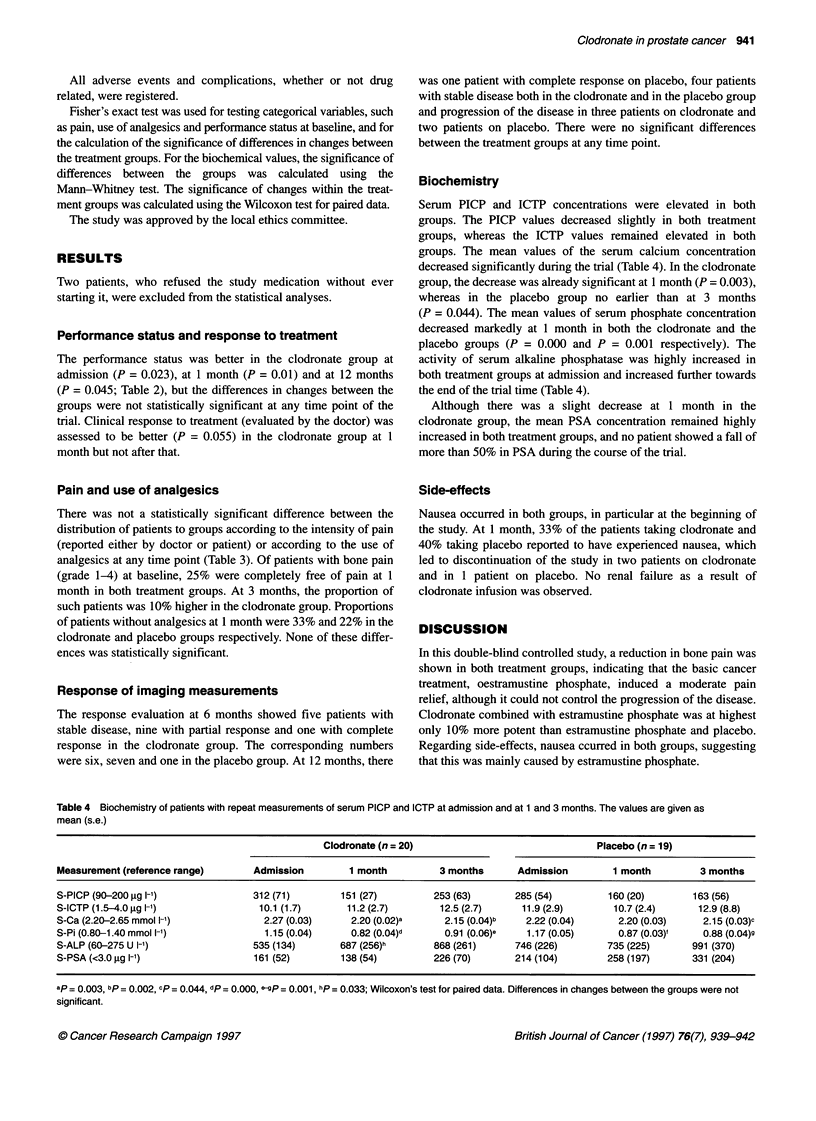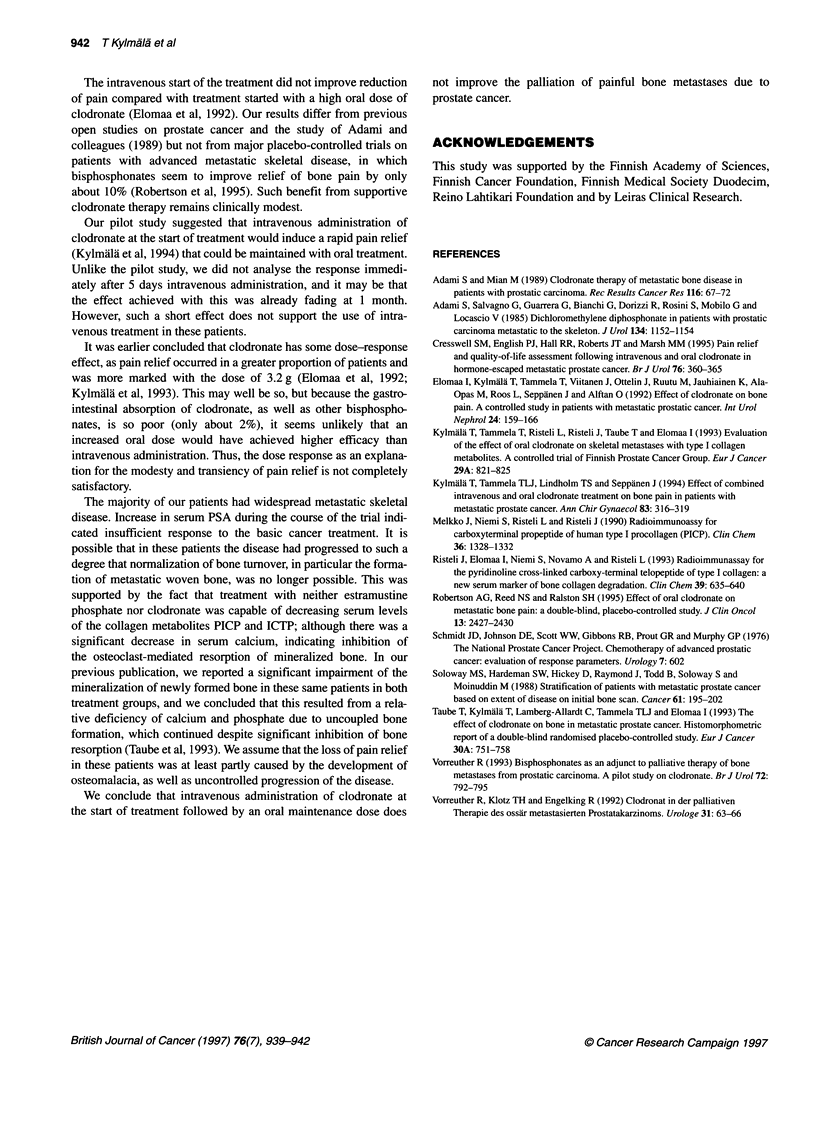# Concomitant i.v. and oral clodronate in the relief of bone pain--a double-blind placebo-controlled study in patients with prostate cancer.

**DOI:** 10.1038/bjc.1997.488

**Published:** 1997

**Authors:** T. KylmÃ¤lÃ¤, T. Taube, T. L. Tammela, L. Risteli, J. Risteli, I. Elomaa

**Affiliations:** Department of Surgery, Tampere University Hospital, Finland.

## Abstract

Fifty-seven patients with advanced prostate cancer resistant to first-line hormonal therapy were treated with estramustine and additionally randomized for treatment with clodronate or placebo. Clodronate treatment was started with 5 days intravenous administration (300 mg day[-1]) and followed by oral treatment (1.6 g day[-1]) for 12 months. Skeletal pain relief was only about 10% better in the clodronate than in the placebo group. The results do not support the superiority of combined intravenous and oral treatment with clodronate compared with oral administration only.


British Journal of Cancer (1997) 76(7), 939–942
© 1997 Cancer Research Campaign

### Concomitant i.v. and oral clodronate in the relief of bone pain – a double-blind placebo-controlled study in patients with prostate cancer

**T Kylmälä[1], T Taube[2], TLJ Tammela[1], L Risteli[3], J Risteli[3] and I Elomaa[2]**

[1]Division of Urology, Department of Surgery, Tampere University Hospital, Finland; [2]Department of Oncology, University of Helsinki, Finland; [3]Departments of Medical Biochemistry and Clinical Chemistry, University of Oulu, Finland

**Summary** Fifty-seven patients with advanced prostate cancer resistant to first-line hormonal therapy were treated with estramustine and additionally randomized for treatment with clodronate or placebo. Clodronate treatment was started with 5 days intravenous administration (300 mg day$^{-1}$) and followed by oral treatment (1.6 g day$^{-1}$) for 12 months. Skeletal pain relief was only about 10% better in the clodronate than in the placebo group. The results do not support the superiority of combined intravenous and oral treatment with clodronate compared with oral administration only.

**Keywords:** prostate cancer; bone metastasis; clodronate; oestramustine phosphate

Only two of the limited number of studies on treatment of painful bone metastases due to prostate cancer with clodronate (Adami et al, 1985; Adami et al, 1989; Elomaa et al, 1992; Vorreuther et al, 1992; Vorreuther, 1993; Kylmälä et al, 1994; Cresswell et al, 1995) have been placebo controlled (Adami et al, 1989; Elomaa et al, 1992). Adami and colleagues (1989) first showed, in 13 patients, that intravenous administration of clodronate was more effective in reducing bone pain than oral administration and that the effect lasted longer when intravenous administration was followed by oral treatment. We have shown that oral treatment with clodronate induces a moderate and transient pain relief in patients with hormone-refractory prostate cancer (Elomaa et al, 1992). It was concluded that the loss of effect resulted partly from dose reduction from 3.2 g to 1.6 g after the first month and partly due to the progression of disease despite basic cancer treatment.

In our recently published open pilot study (Kylmälä et al, 1994), more than half of the patients with prostate cancer and painful bone metastases reported pain relief after 6 days' intravenous administration of clodronate (300 mg day$^{-1}$), and the favourable effect lasted in all but three patients until the follow-up of 3 weeks, when the treatment was continued with oral administration (3.2 g day$^{-1}$).

The present study was conducted to see whether treatment with combined intravenous and oral administration of clodronate would induce a more rapid and effective pain relief than the treatment started with high oral dose. The study was prospective, randomized, double blinded and placebo controlled.

### PATIENTS AND METHODS

The study group comprised 57 prostate cancer patients with progressive metastatic bone disease on bone scan. All patients had failed first-line hormonal treatment. The characteristics of the patients are summarized in Table 1.

The entry criteria demanded oral consent, estimated life expectancy of at least 6 months, no signs of clinically relevant renal or liver insufficiency, no peptic ulcer treated with antacids and no radiation therapy in the 2 weeks preceding the trial. All patients received estramustine phosphate (Estracyt) 280 mg twice

**Table 1** Clinical characteristics of 57 patients with metastatic prostate cancer at base line

| | Clodronate | Placebo |
|---|---|---|
| Total number of patients recruited | 28 | 29 |
| Excluded from statistical analysis | | 2 |
| Mean age (range) (years) | 72 (52–81) | 76 (59–86) |
| Duration of skeletal disease (months) | | |
| Mean (range) | 9 (1–45) | 16 (1–92) |
| Median | 6 | 5 |
| Treatment before the study | | |
| Orchidectomy | 20 | 22 |
| Oestrogen | 5 | 7 |
| LHRH-agonists | 1 | 5 |
| Antiandrogens | 3 | 1 |
| Radiotherapy (to the prostate) | 2 | 0 |
| Performance status (WHO) | | |
| 0 | 3 | 1 |
| 1 | 13 | 5 |
| 2 | 9 | 10 |
| 3 | 3 | 11 |
| 4 | 0 | 0 |
| Bone scintigraphy | | |
| Soloway 1 (< 6) | 9 | 6 |
| Soloway 2 and 3 (≥ 6) | 12 | 14 |
| Soloway 3 (super scan) | 7 | 7 |

LHRH, luteinizing hormone-releasing hormone.

*Received 10 January 1997*
*Revised 1 April 1997*
*Accepted 7 April 1997*

*Correspondence to:* I Elomma, Department of Oncology, University of Helsinki, Haartmaninkatu 4, SF-00290 Helsinki 29, Finland

**Table 2** Performance status by number of patients on clodronate or placebo at each time point

| | Admission* | | 1 month** | | 3 months | | 6 months | | 12 months*** | |
|---|---|---|---|---|---|---|---|---|---|---|
| WHO Classification | Clodronate | Placebo | Clodronate | Placebo | Clodronate | Placebo | Clodronate | Placebo | Clodronate | Placebo |
| 0 | 3 | 1 | 4 | 1 | 6 | 2 | 4 | 2 | 4 | 1 |
| 1 | 13 | 5 | 12 | 7 | 6 | 3 | 4 | 1 | 1 | 2 |
| 2 | 9 | 10 | 6 | 4 | 4 | 6 | 2 | 7 | 0 | 4 |
| 3 | 3 | 11 | 2 | 13 | 3 | 9 | 5 | 1 | 3 | 0 |
| 4 | 0 | 0 | 1 | 1 | 3 | 3 | 2 | 3 | 1 | 1 |

*$P$ = 0.023,**$P$ = 0.01, ***$P$ = 0.045 between clodronate and placebo.

**Table 3** The number of patients according to WHO grading for the intensity of pain (reported by doctor and by patient) and the use of analgesics according to WHO grading scale in both treatment groups at each time point

| | Admission | | 1 month | | 3 months | | 6 months | | 12 months | |
|---|---|---|---|---|---|---|---|---|---|---|
| | Clodronate | Placebo | Clodronate | Placebo | Clodronate | Placebo | Clodronate | Placebo | Clodronate | Placebo |
| Pain (doctor) | | | | | | | | | | |
| 0 | 7 | 1 | 11 | 6 | 9 | 6 | 6 | 5 | 4 | 4 |
| 1 | 11 | 12 | 7 | 9 | 4 | 3 | 3 | 2 | 1 | 2 |
| 2 | 6 | 11 | 4 | 6 | 3 | 9 | 2 | 3 | 2 | 1 |
| 3 | 4 | 2 | 3 | 5 | 4 | 4 | 4 | 3 | 1 | 1 |
| 4 | 0 | 0 | 0 | 0 | 2 | 0 | 2 | 1 | 1 | 0 |
| Pain (patient) | | | | | | | | | | |
| 0 | 6 | 2 | 10 | 6 | 9 | 6 | 6 | 5 | 4 | 4 |
| 1 | 13 | 12 | 9 | 10 | 4 | 5 | 4 | 3 | 1 | 2 |
| 2 | 5 | 12 | 3 | 7 | 2 | 7 | 1 | 5 | 1 | 2 |
| 3 | 3 | 1 | 2 | 3 | 5 | 4 | 4 | 1 | 2 | 0 |
| 4 | 0 | 0 | 0 | 0 | 0 | 0 | 2 | 1 | 0 | 0 |
| Use of analgesics | | | | | | | | | | |
| 0 | 10 | 3 | 13 | 8 | 8 | 5 | 6 | 5 | 4 | 4 |
| 1 | 11 | 13 | 6 | 7 | 6 | 5 | 4 | 3 | 1 | 2 |
| 2 | 6 | 10 | 5 | 9 | 3 | 8 | 3 | 3 | 3 | 2 |
| 3 | 1 | 1 | 1 | 2 | 4 | 4 | 4 | 3 | 1 | 0 |

daily as basic cancer treatment. Clodronate (Bonefos, Leiras) or matched placebo was started with 5 days of intravenous administration (300 mg $day^{-1}$) and was continued by mouth (1.6 g $day^{-1}$) for 12 months.

Pain, performance status and response to treatment were assessed at admission, at 1, 3, 6 and 12 months. The intensity of pain was assessed with a verbal ordinal scale graded from 0 (no pain) to 4 (intolerable pain) by the doctor and with a visual analogue scale (VAS) by the patient. The use of analgesic drugs was evaluated using a four-step grading scale (0, no analgesic drugs; 1, non-narcotic analgesic drugs less than three time per day; 2, non-narcotic analgesic drugs more than three times per day; 3, narcotic analgesic drugs). Performance status was evaluated using a five-step grading scale (0, asymptomatic; 1, minor symptoms; 2, less than 50% of the time in bed; 3, more than 50% of the time in bed; 4, totally bedridden). Clinical response to treatment at each follow-up visit was assessed to be better, same or worse by the doctor.

Scintigraphies were taken at admission and at 6 and 12 months. Response to imaging measurements was evaluated by two investigators independently of each other. The number and extent of hot spots in different sites of the skeleton were recorded according to the Soloway grading scale (Soloway et al, 1988). The criteria for response to treatment were formulated according to the National Prostatic Cancer Project (NPCP; Schmidt et al, 1976) (complete response, initially abnormal bone scan returned to normal; partial response, a reduction of 50% in the number of hot spots and a decrease of at least 50%, in addition to an increase of 25%, at highest, in cross-sectional area of pre-existing lesions; progression, appearance of new hot spots and an increase of more than 25% of the pre-existing lesions).

Serum indices of turnover of type I collagen (the carboxy-terminal propeptide of type I procollagen, PICP, and the pyrdino-line-cross-linked carboxy-terminal telopeptide of type I collagen, ICTP) were analysed at admission and at 1 and 3 months for those patients who survived at least 3 months. The results were examined together with the measurements of serum calcium, phosphate and alkaline phosphatase at the corresponding time points. The radioimmunoassays for analysing the concentrations of PICP and ICTP were performed as described by Melkko et al (1990) and Risteli et al (1993). PICP indicates the formation and ICTP the breakdown of type I collagen.

Response to basic cancer therapy (i.e. estramustine phosphate) was evaluated by monitoring serum concentration of prostate-specific antigen (PSA).

© Cancer Research Campaign 1997

All adverse events and complications, whether or not drug related, were registered.

Fisher's exact test was used for testing categorical variables, such as pain, use of analgesics and performance status at baseline, and for the calculation of the significance of differences in changes between the treatment groups. For the biochemical values, the significance of differences between the groups was calculated using the Mann–Whitney test. The significance of changes within the treatment groups was calculated using the Wilcoxon test for paired data.

The study was approved by the local ethics committee.

### RESULTS

Two patients, who refused the study medication without ever starting it, were excluded from the statistical analyses.

### Performance status and response to treatment

The performance status was better in the clodronate group at admission ($P$ = 0.023), at 1 month ($P$ = 0.01) and at 12 months ($P$ = 0.045; Table 2), but the differences in changes between the groups were not statistically significant at any time point of the trial. Clinical response to treatment (evaluated by the doctor) was assessed to be better ($P$ = 0.055) in the clodronate group at 1 month but not after that.

### Pain and use of analgesics

There was not a statistically significant difference between the distribution of patients to groups according to the intensity of pain (reported either by doctor or patient) or according to the use of analgesics at any time point (Table 3). Of patients with bone pain (grade 1–4) at baseline, 25% were completely free of pain at 1 month in both treatment groups. At 3 months, the proportion of such patients was 10% higher in the clodronate group. Proportions of patients without analgesics at 1 month were 33% and 22% in the clodronate and placebo groups respectively. None of these differences was statistically significant.

### Response of imaging measurements

The response evaluation at 6 months showed five patients with stable disease, nine with partial response and one with complete response in the clodronate group. The corresponding numbers were six, seven and one in the placebo group. At 12 months, there was one patient with complete response on placebo, four patients with stable disease both in the clodronate and in the placebo group and progression of the disease in three patients on clodronate and two patients on placebo. There were no significant differences between the treatment groups at any time point.

### Biochemistry

Serum PICP and ICTP concentrations were elevated in both groups. The PICP values decreased slightly in both treatment groups, whereas the ICTP values remained elevated in both groups. The mean values of the serum calcium concentration decreased significantly during the trial (Table 4). In the clodronate group, the decrease was already significant at 1 month ($P$ = 0.003), whereas in the placebo group no earlier than at 3 months ($P$ = 0.044). The mean values of serum phosphate concentration decreased markedly at 1 month in both the clodronate and the placebo groups ($P$ = 0.000 and $P$ = 0.001 respectively). The activity of serum alkaline phosphatase was highly increased in both treatment groups at admission and increased further towards the end of the trial time (Table 4).

Although there was a slight decrease at 1 month in the clodronate group, the mean PSA concentration remained highly increased in both treatment groups, and no patient showed a fall of more than 50% in PSA during the course of the trial.

### Side-effects

Nausea occurred in both groups, in particular at the beginning of the study. At 1 month, 33% of the patients taking clodronate and 40% taking placebo reported to have experienced nausea, which led to discontinuation of the study in two patients on clodronate and in 1 patient on placebo. No renal failure as a result of clodronate infusion was observed.

### DISCUSSION

In this double-blind controlled study, a reduction in bone pain was shown in both treatment groups, indicating that the basic cancer treatment, oestramustine phosphate, induced a moderate pain relief, although it could not control the progression of the disease. Clodronate combined with estramustine phosphate was at highest only 10% more potent than estramustine phosphate and placebo. Regarding side-effects, nausea ccurred in both groups, suggesting that this was mainly caused by estramustine phosphate.

**Table 4** Biochemistry of patients with repeat measurements of serum PICP and ICTP at admission and at 1 and 3 months. The values are given as mean (s.e.)

| | Clodronate ($n$ = 20) | | | Placebo ($n$ = 19) | | |
|---|---|---|---|---|---|---|
| Measurement (reference range) | Admission | 1 month | 3 months | Admission | 1 month | 3 months |
| S-PICP (90–200 μg $l^{-1}$) | 312 (71) | 151 (27) | 253 (63) | 285 (54) | 160 (20) | 163 (56) |
| S-ICTP (1.5–4.0 μg $l^{-1}$) | 10.1 (1.7) | 11.2 (2.7) | 12.5 (2.7) | 11.9 (2.9) | 10.7 (2.4) | 12.9 (8.8) |
| S-Ca (2.20–2.65 mmol $l^{-1}$) | 2.27 (0.03) | 2.20 (0.02)[a] | 2.15 (0.04)[b] | 2.22 (0.04) | 2.20 (0.03) | 2.15 (0.03)[c] |
| S-Pi (0.80–1.40 mmol $l^{-1}$) | 1.15 (0.04) | 0.82 (0.04)[d] | 0.91 (0.06)[e] | 1.17 (0.05) | 0.87 (0.03)[f] | 0.88 (0.04)[g] |
| S-ALP (60–275 U $l^{-1}$) | 535 (134) | 687 (256)[h] | 868 (261) | 746 (226) | 735 (225) | 991 (370) |
| S-PSA (<3.0 μg $l^{-1}$) | 161 (52) | 138 (54) | 226 (70) | 214 (104) | 258 (197) | 331 (204) |

[a]$P$ = 0.003, [b]$P$ = 0.002, [c]$P$ = 0.044, [d]$P$ = 0.000, [e–g]$P$ = 0.001, [h]$P$ = 0.033; Wilcoxon's test for paired data. Differences in changes between the groups were not significant.

The intravenous start of the treatment did not improve reduction of pain compared with treatment started with a high oral dose of clodronate (Elomaa et al, 1992). Our results differ from previous open studies on prostate cancer and the study of Adami and colleagues (1989) but not from major placebo-controlled trials on patients with advanced metastatic skeletal disease, in which bisphosphonates seem to improve relief of bone pain by only about 10% (Robertson et al, 1995). Such benefit from supportive clodronate therapy remains clinically modest.

Our pilot study suggested that intravenous administration of clodronate at the start of treatment would induce a rapid pain relief (Kylmälä et al, 1994) that could be maintained with oral treatment. Unlike the pilot study, we did not analyse the response immediately after 5 days intravenous administration, and it may be that the effect achieved with this was already fading at 1 month. However, such a short effect does not support the use of intravenous treatment in these patients.

It was earlier concluded that clodronate has some dose–response effect, as pain relief occurred in a greater proportion of patients and was more marked with the dose of 3.2 g (Elomaa et al, 1992; Kylmälä et al, 1993). This may well be so, but because the gastrointestinal absorption of clodronate, as well as other bisphosphonates, is so poor (only about 2%), it seems unlikely that an increased oral dose would have achieved higher efficacy than intravenous administration. Thus, the dose response as an explanation for the modesty and transiency of pain relief is not completely satisfactory.

The majority of our patients had widespread metastatic skeletal disease. Increase in serum PSA during the course of the trial indicated insufficient response to the basic cancer treatment. It is possible that in these patients the disease had progressed to such a degree that normalization of bone turnover, in particular the formation of metastatic woven bone, was no longer possible. This was supported by the fact that treatment with neither estramustine phosphate nor clodronate was capable of decreasing serum levels of the collagen metabolites PICP and ICTP; although there was a significant decrease in serum calcium, indicating inhibition of the osteoclast-mediated resorption of mineralized bone. In our previous publication, we reported a significant impairment of the mineralization of newly formed bone in these same patients in both treatment groups, and we concluded that this resulted from a relative deficiency of calcium and phosphate due to uncoupled bone formation, which continued despite significant inhibition of bone resorption (Taube et al, 1993). We assume that the loss of pain relief in these patients was at least partly caused by the development of osteomalacia, as well as uncontrolled progression of the disease.

We conclude that intravenous administration of clodronate at the start of treatment followed by an oral maintenance dose does not improve the palliation of painful bone metastases due to prostate cancer.

### ACKNOWLEDGEMENTS

This study was supported by the Finnish Academy of Sciences, Finnish Cancer Foundation, Finnish Medical Society Duodecim, Reino Lahtikari Foundation and by Leiras Clinical Research.

### REFERENCES

Adami S and Mian M (1989) Clodronate therapy of metastatic bone disease in patients with prostatic carcinoma. *Rec Results Cancer Res* **116**: 67–72

Adami S, Salvagno G, Guarrera G, Bianchi G, Dorizzi R, Rosini S, Mobilo G and Locascio V (1985) Dichloromethylene diphosphonate in patients with prostatic carcinoma metastatic to the skeleton. *J Urol* **134**: 1152–1154

Cresswell SM, English PJ, Hall RR, Roberts JT and Marsh MM (1995) Pain relief and quality-of-life assessment following intravenous and oral clodronate in hormone-escaped metastatic prostate cancer. *Br J Urol* **76**: 360–365

Elomaa I, Kylmälä T, Tammela T, Viitanen J, Ottelin J, Ruutu M, Jauhiainen K, Ala-Opas M, Roos L, Seppänen J and Alftan O (1992) Effect of clodronate on bone pain. A controlled study in patients with metastatic prostatic cancer. *Int Urol Nephrol* **24**: 159–166

Kylmälä T, Tammela T, Risteli L, Risteli J, Taube T and Elomaa I (1993) Evaluation of the effect of oral clodronate on skeletal metastases with type I collagen metabolites. A controlled trial of Finnish Prostate Cancer Group. *Eur J Cancer* **29A**: 821–825

Kylmälä T, Tammela TLJ, Lindholm TS and Seppänen J (1994) Effect of combined intravenous and oral clodronate treatment on bone pain in patients with metastatic prostate cancer. *Ann Chir Gynaecol* **83**: 316–319

Melkko J, Niemi S, Risteli L and Risteli J (1990) Radioimmunoassy for carboxyterminal propeptide of human type I procollagen (PICP). *Clin Chem* **36**: 1328–1332

Risteli J, Elomaa I, Niemi S, Novamo A and Risteli L (1993) Radioimmunassay for the pyridinoline cross-linked carboxy-terminal telopeptide of type I collagen: a new serum marker of bone collagen degradation. *Clin Chem* **39**: 635–640

Robertson AG, Reed NS and Ralston SH (1995) Effect of oral clodronate on metastatic bone pain: a double-blind, placebo-controlled study. *J Clin Oncol* **13**: 2427–2430

Schmidt JD, Johnson DE, Scott WW, Gibbons RB, Prout GR and Murphy GP (1976) The National Prostate Cancer Project. Chemotherapy of advanced prostatic cancer: evaluation of response parameters. *Urology* **7**: 602

Soloway MS, Hardeman SW, Hickey D, Raymond J, Todd B, Soloway S and Moinuddin M (1988) Stratification of patients with metastatic prostate cancer based on extent of disease on initial bone scan. *Cancer* **61**: 195–202

Taube T, Kylmälä T, Lamberg-Allardt C, Tammela TLJ and Elomaa I (1993) The effect of clodronate on bone in metastatic prostate cancer. Histomorphometric report of a double-blind randomised placebo-controlled study. *Eur J Cancer* **30A**: 751–758

Vorreuther R (1993) Bisphosphonates as an adjunct to palliative therapy of bone metastases from prostatic carcinoma. A pilot study on clodronate. *Br J Urol* **72**: 792–795

Vorreuther R, Klotz TH and Engelking R (1992) Clodronat in der palliativen Therapie des ossär metastasierten Prostatakarzinoms. *Urologe* **31**: 63–66

*© Cancer Research Campaign 1997*